# Sphinx: merging knowledge-based and *ab initio* approaches to improve protein loop prediction

**DOI:** 10.1093/bioinformatics/btw823

**Published:** 2017-01-16

**Authors:** Claire Marks, Jaroslaw Nowak, Stefan Klostermann, Guy Georges, James Dunbar, Jiye Shi, Sebastian Kelm, Charlotte M Deane

**Affiliations:** 1 Department of Statistics, University of Oxford, Oxford, UK; 2 Pharma Research and Early Development, Informatics, Roche Innovation Center Munich, Penzberg, DE, Germany; 3 Pharma Research and Early Development, Large Molecule Research, Roche Innovation Center Munich, Penzberg, DE, Germany; 4 Department of Informatics, UCB Pharma, Slough, UK

## Abstract

**Motivation:**

Loops are often vital for protein function, however, their irregular structures make them difficult to model accurately. Current loop modelling algorithms can mostly be divided into two categories: knowledge-based, where databases of fragments are searched to find suitable conformations and *ab initio*, where conformations are generated computationally. Existing knowledge-based methods only use fragments that are the same length as the target, even though loops of slightly different lengths may adopt similar conformations. Here, we present a novel method, Sphinx, which combines *ab initio* techniques with the potential extra structural information contained within loops of a different length to improve structure prediction.

**Results:**

We show that Sphinx is able to generate high-accuracy predictions and decoy sets enriched with near-native loop conformations, performing better than the *ab initio* algorithm on which it is based. In addition, it is able to provide predictions for every target, unlike some knowledge-based methods. Sphinx can be used successfully for the difficult problem of antibody H3 prediction, outperforming RosettaAntibody, one of the leading H3-specific *ab initio* methods, both in accuracy and speed.

**Availability and Implementation:**

Sphinx is available at http://opig.stats.ox.ac.uk/webapps/sphinx.

**Supplementary information:**

Supplementary data are available at *Bioinformatics* online.

## 1 Introduction

Loops, the irregular regions of a protein that connect secondary structure elements, often play a vital functional role. Structural information is critical to fully understanding a protein’s properties – however, obtaining structures experimentally is time-consuming and difficult, especially for loop regions, which can be missing from experimentally solved structures (Petoukhov *et al.*, 2002). Due to the variability of loop structures and sequences between homologues, predicting loop conformations remains challenging – loops are usually the least accurate regions of a protein model (Baker and Sali, 2001; Gront *et al.*, 2012).

Traditionally, methods for predicting protein loop structures are divided into two categories, knowledge-based or *ab initio*, depending on how they generate possible conformations (decoys). Knowledge-based methods rely upon databases of previously observed protein structure fragments. Loop structures are selected according to certain criteria such as fragment length (i.e. they must be the same length as the target loop), fragment-target sequence similarity and how closely the anchor geometry of the fragment matches that of the target loop. Methods of this type are extremely fast and can be very accurate when the structure of the target loop is similar to one previously observed. However, there is not currently enough structural data to cover the conformational space, especially for long loops (Fernandez-Fuentes and Fiser, 2006). When a similar loop structure has not been observed previously, knowledge-based methods either give poor predictions or fail to return a prediction at all. Examples of this type of algorithm include FREAD (Choi and Deane, 2010; Deane and Blundell, 2001), SuperLooper (Hildebrand *et al.*, 2009), LoopWeaver (Holtby *et al.*, 2013), LIP (Michalsky *et al.*, 2003) and LoopIng (Messih *et al.*, 2015).

*Ab initio* methods do not rely on previously observed structures; instead, decoys are produced computationally. *Ab initio* methods work by exploring the possible conformational space, for example by randomly sampling the ϕ and *ψ* dihedral angles of the loop. Depending on how the loops are built, the continuity of the protein backbone may need to be enforced through the implementation of a closure algorithm, such as cyclic coordinate descent (CCD) (Canutescu and Dunbrack, 2003), random tweak (Shenkin *et al.*, 1987) or KIC (Mandell *et al.*, 2009). Like knowledge-based methods, *ab initio* algorithms also have their limitations: they are computationally expensive, since many decoys must be generated to sample the conformational space sufficiently; and their prediction accuracy decreases with loop length (as the number of degrees of freedom increases). *Ab initio* methods include PLOP (Jacobson *et al.*, 2004), MODELLER (Fiser *et al.*, 2000), Loopy (Xiang *et al.*, 2002), LoopBuilder (Soto *et al.*, 2007), LEAP (Liang *et al.*, 2014), and the loop modelling routine within Rosetta (Stein and Kortemme, 2013).

The idea of a hybrid loop modelling algorithm, combining knowledge-based and *ab initio* approaches, has been explored before. CODA (Deane and Blundell, 2001) generates decoys using a knowledge-based method and an *ab initio* method separately, then combines the two decoy sets and makes a consensus prediction. Fasnacht *et al.* (2014), Martin *et al.* (1989), and Whitelegg and Rees (2000)) have used similar approaches and applied it to modelling the complementarity determining regions (CDRs) of antibodies – initial conformations are selected from a database of structures, and the middle section is then remodelled using *ab initio* techniques. In all of these examples, however, the *ab initio* and knowledge-based sections of the algorithms are kept distinct from one another. An alternative approach using Rosetta is described by Rohl *et al.*, 2004 – this used a Monte Carlo-based fragment assembly method, in conjunction with a minimization protocol. The fragments used by this algorithm are very short (three residues for loops under 15 residues in length) and therefore any structural similarity is only considered for short segments of the target loop, not all of it.

Recent research into the canonical conformations of antibody CDRs has shown that loops of different lengths can adopt similar structures (Nowak *et al.*, 2016), indicating that loops with a different number of residues could still provide useful information when modelling a particular target loop. However, no existing methods are able to use this knowledge, since they either do not use previously known structures, must only use length-matched fragments, or build conformations by assembling very short fragments. Some algorithms, such as Frag’r’us (Bonet *et al.*, 2014), allow the user to remodel loop structures using fragments of a different length to the original loop, however, the fragment must still be of the same length as the desired target loop. Here, we introduce a new loop modelling method, Sphinx, that can use this extra source of structural information, by integrating aspects of both knowledge-based and *ab initio* approaches in a novel way. Sphinx begins the loop modelling process by identifying potential fragments that are slightly shorter than the loop to be predicted. By combining the structural information from such fragments with *ab initio* techniques, the length is adjusted and decoys of the correct length are generated. In this way, we are able to use the additional information present in different length loops. Sphinx is not limited to predicting what has already been observed, like a traditional knowledge-based method, nor does it ignore the available structural information, as would be the case using a purely *ab initio* approach. Our algorithm gives a prediction in every case (unlike some knowledge-based methods) and generates decoy sets that are enriched with near-native conformations. This leads to high-accuracy predictions, demonstrated by its ability to outperform existing software in the challenging problem of in antibody CDR H3 prediction.

## 2 Methods

**Fig. 1.**
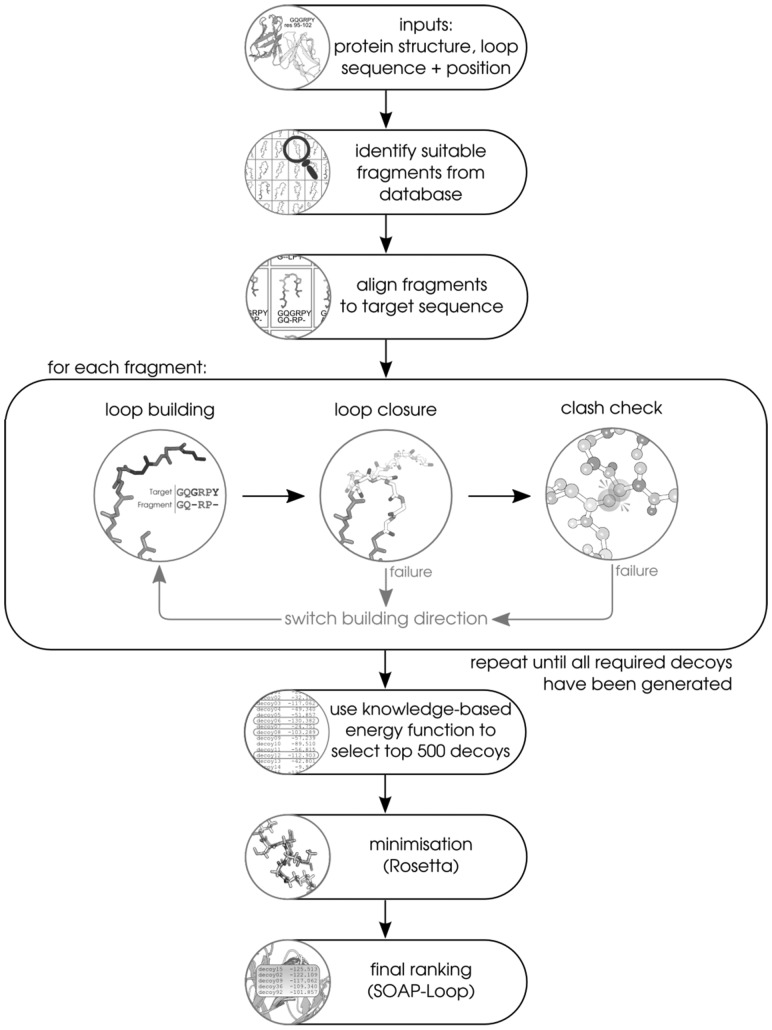
Flowchart describing the Sphinx algorithm. As input, the algorithm requires the protein structure on which the loop is to be modelled, and the loop sequence and location. The algorithm begins by searching through a database of loop fragments. Suitable fragments, that are shorter than the target loop, are used to help build a set of 100 decoys. The number of fragments used depends on the length (*n*) of the target – 12.5(n−2) for even lengths and 12.5(n−1) for odd lengths. For any residues aligned between the target and the fragment, structural information is copied across. Any residues not aligned to fragment residues are built *ab initio*. Each loop structure undergoes a closure step and a clash check – if closure fails or there are clashes, the next decoy is built from the alternate anchor residue (‘switching’). Once all decoys have been generated, a loop-specific energy function is used to select the top 500 decoys, which subsequently undergo minimization (using Rosetta) and final ranking (with SOAP-Loop). A larger, colour version of this figure is provided in the Supplementary Information

The Sphinx algorithm is a combination of FREAD (Choi and Deane, 2010; Deane and Blundell, 2001), a knowledge-based method and our own *ab initio* algorithm. The inputs to Sphinx are a protein structure or model (in PDB format) and the location and sequence of the loop to be modelled. The algorithm includes four main steps: database search, loop building, loop closure and decoy selection. A flowchart of the algorithm is given in Figure 1.

### 2.1 Database search

The first step of the Sphinx algorithm is the search of a fragment database, for fragments that are a different length to the target, but that may share the same conformation. To create this database, the loop structures of all PDB entries with non-identical sequences [according to PISCES (Wang and Dunbrack, 2003), using the structure with the best resolution for each sequence], plus three residues on each side, were extracted and split into all possible fragments of 3–30 residues in length. Loop regions were located using DSSP (Joosten *et al.*, 2011) and were defined as regions connecting secondary structures of at least three residues in length. This led to a database containing the loop structures of 65 108 protein chains (created October 2015).

Fragments from the database are selected as potential starting points for prediction depending on three criteria:

1. length – the fragment must be shorter than the target loop, since preliminary tests indicated that fragments longer than the target were not able to produce decoys of the same quality. This may be because shorter loops are represented more in the database.
2. the anchor geometry of the fragment (the two residues on each side of the loop) must match that of the target. As is the case in the FREAD algorithm (Choi and Deane, 2010; Deane and Blundell, 2001), the distances between the C*α* atoms of the anchor residues (the two residues on each side of the loop) are used to select fragments with similar anchor geometry.
3. sequence similarity – the sequences of fragments that meet the first two criteria are aligned to the target sequence using the Needleman–Wunsch algorithm (Needleman and Wunsch, 1970). These alignments are scored using an environment-specific substitution (ESS) score (a score which takes into account the dihedral angles of the fragment residues) (Deane and Blundell, 2001; Hill *et al.*, 2011), as implemented in FREAD.

The best fragments according to their ESS scores are then passed to the loop building stage of the algorithm, where their length (n) will be adjusted using *ab initio* techniques. Based on exploratory testing of Sphinx (using the general loop set described in Section 2.6), the number and length of fragments to be used depend on the following rules:

$$\text{minimum}\;\text{target}\;\text{length}=\{\begin{array}{cc} n-3 & \;\text{if}\;n\leq 12\\ n-4 & \;\text{if}\;n>12 \end{array}$$

$$\;\text{number}\;\text{of}\;\text{fragments}=\{\begin{array}{cc} 12.5(n-2) & \;\text{if}\;n\;\text{is}\;\text{even}\;\\ 12.5(n-1) & \;\text{if}\;n\;\text{is}\;\text{odd}\; \end{array}$$

Throughout this work, we ignore fragments that come from sequence-identical chains to the target protein, as in a real modelling situation these structures would not be available.

### 2.2 Loop building

The structure and sequence alignment information of each selected fragment is then used to build a set of 100 complete loop decoys of the correct length. Each decoy is built, one residue at a time, onto one of the anchor residues, using the algorithm described by Parsons *et al.*, 2005.

For target residues that are paired with a fragment residue in the sequence alignment, the structural data (bond lengths, bond angles and dihedral angles) used to build the residue are calculated from the corresponding fragment residue. For target residues that are not aligned to a fragment residue, the structural data are randomly selected from a set of purpose-built distributions. Bond lengths and angles are drawn at random from the Gaussian distributions (parameters given by Engh and Huber, 1991), and dihedral (ϕ/*ψ*) angles are selected from a set of residue-specific Ramachandran distributions, built using the same set of loop structures that were used to create the fragment database (see previous section).

### 2.3 Loop closure

Since the termini of the loop must be connected to the anchor residues to form a continuous backbone, the structure generated must be ‘closed’; for example, if the loop was built from the N-anchor (the anchor nearest to the N terminus of the protein), then the structure must be altered so that the other end of the loop attaches to the C-anchor. The loop closure method used by Sphinx is CCD (Canutescu and Dunbrack, 2003), which works by iteratively changing the ϕ/*ψ* angles of the loop, gradually minimizing the distance between the free end of the loop and the anchor.

For Sphinx, we have written a modified version of the CCD algorithm (as suggested by Canutescu and Dunbrack, 2003), where constraints are put on the angle changes so that only the allowed regions of the Ramachandran plot are occupied (see Supplementary Information for details). Thus, all decoys generated by Sphinx will be physically feasible.

It may be preferential for a loop to be built from one particular anchor residue instead of the other. Therefore, if loop closure fails, or the resulting loop structure causes clashes between atoms in the structure, then the loop decoy is discarded and the next loop decoy is built from the alternate anchor residue. This approach, known as ‘switching’ (Chys and Chacon, 2013) allows the preferred direction of loop building to dominate.

Once 100 decoys have been made from a fragment (or the number of failures reaches a cut-off value of 3000), the algorithm moves onto the next fragment until all selected fragments have been used.

### 2.4 Decoy selection

Once a complete set of decoys has been generated (100 per fragment), the number is reduced to 500 using our own loop-specific, knowledge-based energy function. This function is based on the RAPDF (Samudrala and Moult, 1998) and compares the distances between atoms of real structures to those in a decoy. Our version considers only backbone and C_β_ atoms, meaning that the time-consuming process of side-chain addition does not have to be carried out. The data for this function were calculated from a non-redundant set of structures (with a maximum sequence identity of 40% and a resolution cut-off of 2 Å), considering only those pairwise distances involving loop atoms. The function uses six bins for the data (one bin for distances between 0 and 3 Å, followed by five 1 Å bins up to a maximum of 8 Å).

Once the top 500 decoys have been selected, side-chains are added using SCWRL4 (Krivov *et al.*, 2009), they are minimized using the loop refinement protocol within Rosetta (see Supplementary Information) and subsequently ranked using the SOAP-Loop potential in MODELLER (Dong *et al.*, 2013), a statistical potential developed specifically for loop modelling. SOAP-Loop is fast and gave the best results when tested against other ranking methods (see Supplementary Information).

### 2.5 Sphinx-H3

The H3-specific version of Sphinx is different to the general version in three ways; the database of fragments searched, the dihedral data used and the number of fragments from the database to be used in loop building.

The H3-specific database was created by extracting the structures of all the H3 loops that were present in the Structural Antibody Database (SAbDab) (Dunbar *et al.*, 2014), which were then split into all their possible fragments (in the same way as the general fragment database). We define the H3 loop region according to Chothia and Lesk (1987). In the Chothia numbering scheme, the H3 is found on the heavy chain between residues 95 and 102 inclusive. At the time, the database was made (April 2015), this led to a database containing all possible fragments of 3043 H3 structures, from 1848 different PDB entries.

As there are currently not enough H3 structures available to produce residue-specific Ramachandran distributions, we constructed the distributions using a novel resampling method that combines general protein loop and H3 data (see Supplementary Information for details). The resampling method makes the Ramachandran distributions less sparsely populated while maintaining the unique nature of the H3 data.

To verify the ability of Sphinx to predict H3 loop structures, we first ran it on a target set of H3 loops (see Supplementary Information for details). Following this test, we allowed Sphinx to use twice as many fragments in loop building than were used in the case of general protein loops.

### 2.6 Datasets

Three datasets are used in this paper: a general protein loop set and two sets of antibody H3 loops. From the general protein loops that were extracted to make the fragment database, a series of loops were selected with no more than 40% sequence identity, resolution below 2 Å, no B-factor above 30, and no hydrogen bonds to the surrounding crystal. From these loops, a dataset of 36 targets was selected, with lengths ranging from 6 to 18 residues. The first set of antibody H3 targets was taken from Sivasubramanian *et al.* (2009). The target 2ai0 was removed from the original set since the structure is incomplete and was previously incorrectly designated as a 6-residue loop. Loops that interact with other proteins in the crystal were also removed, leading to a set of 39 targets which range in length from 4 to 22 residues. The second set of H3 targets are the ten targets from the second round of the Antibody Modelling Assessment II. Complete lists of targets are given in the Supporting Information.

## 3 Results

Sphinx begins its prediction by searching for fragments of a slightly different length but possibly a similar conformation to the target loop, as indicated by sequence similarity and anchor geometry. By using this knowledge-based approach in tandem with *ab initio* techniques, these fragments can be adjusted to be the correct length. In all cases, fragments from protein chains that are identical in sequence to the target chain, and fragments of the same length as the target that could be found using a normal knowledge-based method, are ignored. All RMSDs are calculated after first superimposing the rest of the protein structure, or in the case of the antibody models, the framework residues of the heavy chain [as defined in the second Antibody Modelling Assessment (Teplyakov *et al.*, 2014)]. All reported RMSDs use all backbone atoms of the loop, except for the Anitbody Modelling Assessment II target set, which uses only the carbonyl carbon and oxygen atoms of residues 95–100*x* (using the Chothia numbering scheme).

### 3.1 Comparison to knowledge-based and *ab initio* methods

**Fig. 2.**
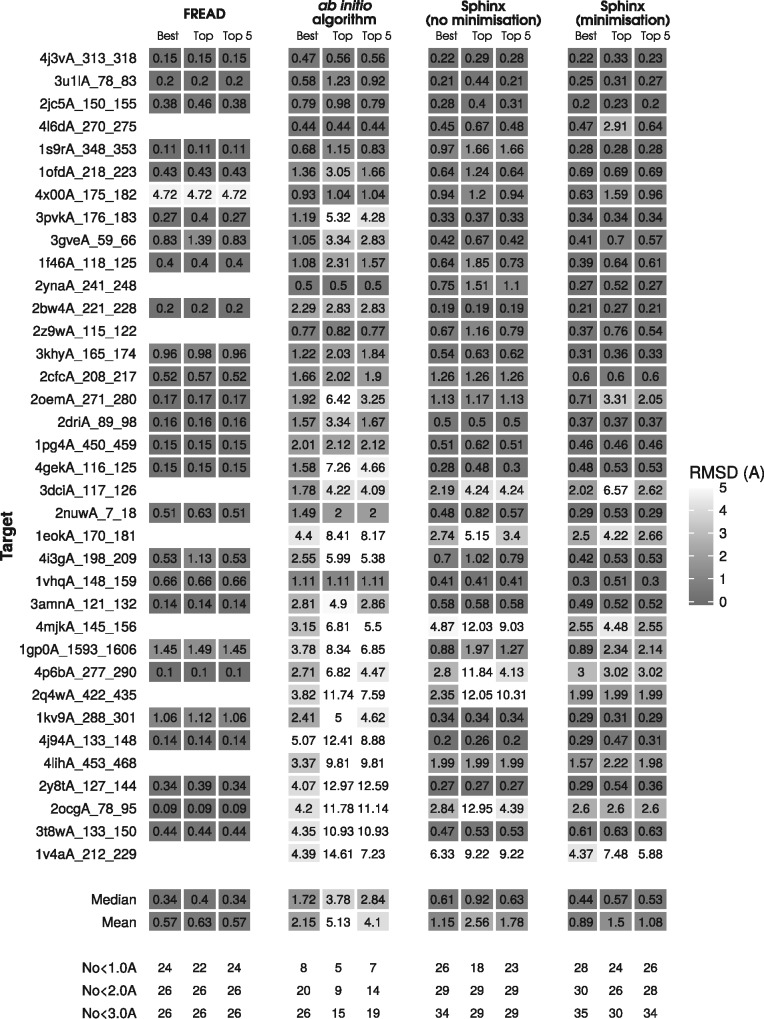
Performance of Sphinx compared to its constituent knowledge-based and *ab initio* parts, for general protein loop types. Targets are listed in order of increasing length. ‘Best’ refers to the decoy in the set that is closest to the native structure, when considering the 500 decoys returned by the loop-specific energy function (for Sphinx and the *ab initio* method) or all the returned decoys (for FREAD). ‘Top’ means the decoy that is predicted to be the best, and ‘Top 5’ denotes the RMSD of the best decoy amongst the five top-ranked. A colour version of this figure can be found in the Supplementary Information

Figure 2 shows the accuracy of the methods on general loops. The methods shown are Sphinx and the algorithms on which it is based: the knowledge-based method FREAD (Choi and Deane, 2010; Deane and Blundell, 2001) and our own *ab initio* method. FREAD is a knowledge-based method, which searches a database for suitable fragments of the same length as the target. We ran FREAD using the same fragment database as used by Sphinx (Section 2), which on average returned seven decoys per target. The *ab initio* method can be thought of as running Sphinx without any fragment information – i.e. all bond lengths, angles and dihedral angles are chosen randomly from the relevant distributions. To ensure a fair comparison, the *ab initio* method was run so as to generate the same number of decoys for each target as Sphinx (around 11 000 on average), and its decoys were ranked in the same way. The results in Figure 2 consider only the 500 decoys selected by our loop specific energy function and the top decoys are selected with SOAP-Loop, either before or after minimization with Rosetta.

**Fig. 3.**
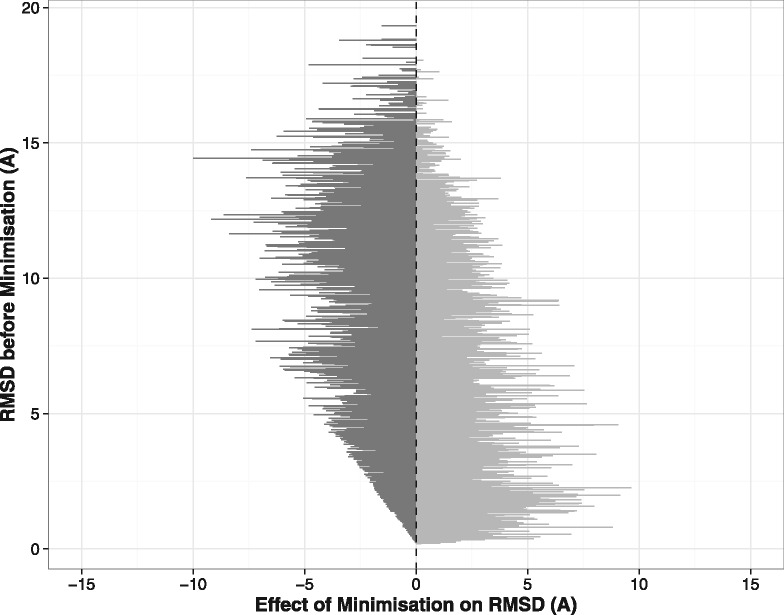
The effect of Rosetta minimization on decoy RMSD, for the general loop target set. The change in RMSD of each decoy is represented as a horizontal line – the position of the line on the y axis indicates the RMSD before minimization, while the length of the line represents the change in RMSD. Decoys with improved RMSDs are on the left of the figure in dark grey while those whose RMSDs were made worse by the minimization step are on the right

The effect of the Rosetta minimization protocol on decoy RMSD is shown in Figure 3. It can be seen that the refinement procedure does not consistently move the decoy structures closer to the native conformation. Overall, 59.1% of decoys have lower RMSDs after minimization, with an average improvement of 0.97 Å. Decoys that before minimization had a high RMSD are more likely to be improved by the minimization protocol; 60% of decoys that begin with an RMSD of over 1.5 Å are improved (with an average reduction in RMSD of 1.12 Å), while 55% of those with RMSDs below 1.5 Å are moved closer to the native structure (with an average RMSD improvement of 0.31 Å).

However, even though the minimization does not reliably improve RMSDs, overall the results are improved by its inclusion (Fig. 2). This appears to be due to SOAP-Loop’s enhanced ability to select near-native decoys – the mean RMSD of the top-ranked decoy is reduced from 2.56 to 1.50 Å, and the mean RMSD of the best decoy in the top five decreased from 1.78to 1.08 Å. In addition, the number of sub-angstrom top-ranked predictions is increased from 18 to 24.

Figure 2 shows that FREAD is more accurate than Sphinx on average, but there are some targets (9) for which FREAD failed to find suitable fragments in the database. Sphinx, on the other hand, returned a prediction in every case. In addition, for 20 of the 36 targets Sphinx was able to generate a better decoy than FREAD, and for 28 targets, Sphinx produced a decoy with an RMSD of below 1 Å; four more than FREAD. Sphinx also gives more top-ranked decoys with sub-angstrom RMSDs, with 24 compared to 22 for FREAD.

Sphinx, by including information from fragments, is able to considerably outperform its base *ab initio* algorithm. When considering the top 500 decoys, Sphinx is able to generate more accurate conformations – the average RMSD of the best decoy produced was 0.89 Å, compared to 2.15 Å for the *ab initio* algorithm, and overall the lowest-RMSD decoy generated by Sphinx was better than that of the *ab initio* algorithm in 33 cases. The *ab initio* algorithm could only produce a conformation with an RMSD below 1 Å for 8 targets; Sphinx gave 28.

**Fig. 4.**
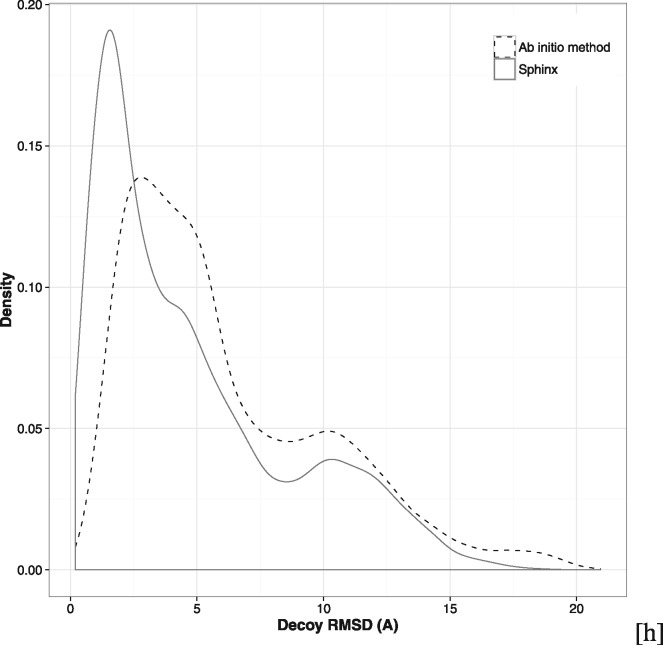
RMSD distributions for the 500-decoy sets produced by Sphinx and its base *ab initio* method. Decoy sets produced by Sphinx contain proportionally more near-native decoys. Data shown is for all targets in the general loop dataset

The improved ability of Sphinx to produce near-native loop conformations is further illustrated by the RMSD distributions of the decoy sets produced (Fig. 4). There is a greater proportion of low-RMSD decoys in the decoy set generated by Sphinx, indicating that the inclusion of fragment information helps to direct the conformational search towards near-native structures.

The RMSDs of the top-ranked decoys (as predicted by SOAP-Loop) are significantly better when using Sphinx compared to the base *ab initio* method, with an average RMSD of 1.50 Å compared to 5.13 Å for the *ab initio* algorithm. The five top-ranked decoys contained 26 and 7 sub-angstrom conformations for Sphinx and the *ab initio* method, respectively. The improved accuracy is especially noticeable for longer loops – for the 16 targets of 12 or more residues, the *ab initio* method gave no sub-angstrom predictions, while Sphinx was able to produce 7.

**Fig. 5.**
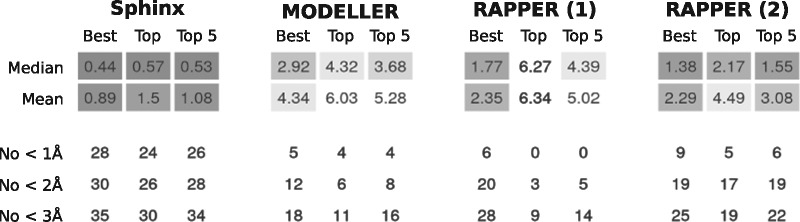
Comparison of Sphinx to other *ab initio* methods: the loop modelling algorithm of MODELLER and RAPPER. RAPPER results are shown using both the method’s own ranking system (1) and the ranking procedure used by Sphinx (2)

To compare Sphinx’s performance to other *ab initio* algorithms, we used the loop modelling algorithm within the MODELLER software (Fiser *et al.*, 2000) as well as RAPPER (DePristo *et al.*, 2003; de Bakker *et al.*, 2003) to model the loops in the general target set. In accordance with their respective papers, for MODELLER we generated 500 decoys for each target, and for RAPPER we generated 1000. As well as using each method’s scoring system to rank the decoys, for RAPPER we also used the Sphinx ranking protocol (see Section 2.4). The results of this comparison are shown in Figure 5. Sphinx is better than the other two methods by every measure. The decoy sets produced by Sphinx are of a higher quality, with an average best RMSD of 0.89 Å compared to 4.34, 2.35 and 2.29 Å for MODELLER, RAPPER and RAPPER using our ranking system, respectively. Sub-ångström decoys were generated by Sphinx for most of the targets (28), while this was rare for the other methods. Considering the top-ranked decoy for each target, Sphinx’s predictions are considerably more accurate, with an average RMSD of 1.50 Å compared to 6.03 Å, 6.34 Å and 4.49 Å for the other three algorithms. The use of Sphinx’s ranking procedure on the decoys generated by RAPPER improved the latter’s performance, for example, decreasing the average top RMSD by 1.85 Å, but Sphinx’s enhanced ability to sample near-native conformations means that its accuracy is still higher. It should be noted, however, that since members of the general loop set were used to set parameters used by Sphinx, this test may overstate its performance.

### 3.2 Antibody H3 prediction

One type of protein for which loop modelling is key is antibodies – a class of protein produced by the immune system to bind to and initiate the removal of foreign substances. Their exceptional ability to bind to a huge variety of antigens, with high specificity and affinity, makes them ideally suited for use as therapeutics (Chames *et al.*, 2009).

The region of the antibody that both contributes the most to antibody binding and introduces the most diversity is the H3 loop (the third CDR of the heavy chain) – it is found in the centre of the binding site, forms the most contacts with the antigen (Alzari *et al.*, 1988; MacCallum *et al.*, 1996) and has the greatest effect on the energetics of binding (Kunik and Ofran, 2013). Knowledge of H3 structures is therefore extremely useful, enabling predictions to be made about antibody binding properties and hence their suitability as drugs (Clark *et al.*, 2006; Diskin *et al.*, 2011; Kiyoshi *et al.*, 2014; Kuroda *et al.*, 2012; Krawczyk *et al.*, 2013; Lewis *et al.*, 2014; Lippow *et al.*, 2007; Thakkar *et al.*, 2014).

Unfortunately, H3 structure prediction is a particularly difficult problem, as demonstrated by the results of a recent blind prediction study (Teplyakov *et al.*, 2014), where the other five CDRs (L1, L2, L3, H1 and H2) were predicted with an average RMSD of 1.2, 0.5, 1.6, 1.1 and 1.0 Å respectively, while the H3 loop was predicted with an average RMSD of 3.5 Å. The difficulty arises due to the diversity of H3 structures, which is caused by gene recombination and junctional diversification (Jeske *et al.*, 1984; Tonegawa, 1983). H3 lengths are extremely variable and tend to be longer than general protein loops (Li, 2013), meaning that searching the entire possible conformational space is challenging. The use of H3-specific methods can help address this problem, however, there are currently less than 2500 antibody structures in the PDB (Dunbar *et al.*, 2014), each containing only one H3 loop, or a few copies of the same loop. Given the sequence and length diversity of H3, this covers only a marginal fraction of possible conformations. The challenging nature of the problem, however, makes H3 an ideal loop on which to test the performance of loop modelling algorithms (Sellers *et al.*, 2010).

**Fig. 6.**
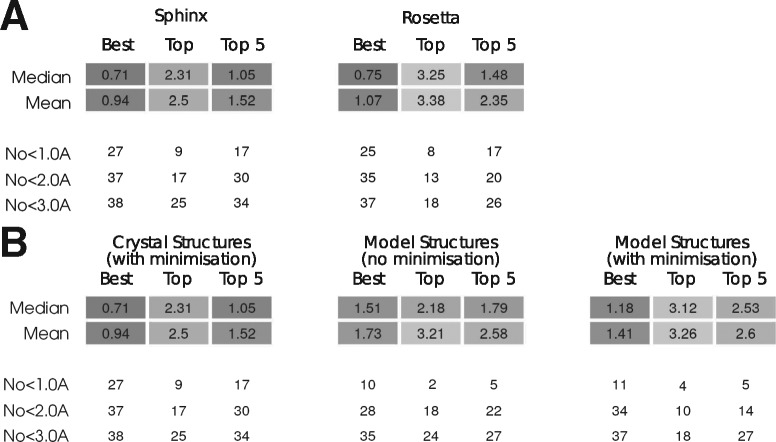
Sphinx-H3 prediction accuracy. (**A**) Comparison of Sphinx and Rosetta antibody H3 loop prediction. (**B**) Performance of Sphinx antibody H3 loop prediction in a non-native environment

We produced an H3-specific version of our algorithm, Sphinx-H3 (see Section 2 for details) and used it to predict the structures of 39 H3 loops, taken from the set used to test RosettaAntibody (Sivasubramanian *et al.*, 2009). To compare our results to other software, we also generated predictions using the H3 modelling routine within RosettaAntibody; a leading H3-specific *ab initio* loop prediction algorithm. This uses the next-generation kinematic modelling (KIC) approach described by Stein and Kortemme (2013) – the full command used is given in the Supplementary Information. The decoys obtained using Rosetta were ranked using its internal scoring function. We generated 500 decoys per target, like in Stein and Kortemme (2013), and the same number from Sphinx that undergo minimization and final ranking. This allows us to compare execution times. Predictions were generated with the rest of the antibody in its native conformation, in the same manner as the second round of the second Antibody Modelling Assessment (Teplyakov *et al.*, 2014). Figure 6A shows the results of this comparison.

The ability of Sphinx-H3 to generate near-native H3 conformations is comparable to RosettaAntibody: the average RMSD of the best decoy generated is 0.94 Å (median 0.71 Å) for Sphinx-H3 compared to 1.07 Å (median 0.75 Å) for RosettaAntibody. After decoy ranking, Sphinx-H3 outperforms RosettaAntibody, producing an average top-ranked RMSD of 2.5 Å (median 2.31 Å) compared to 3.38 Å (median 3.25 Å), and giving predictions below 2 Å for 17 targets (compared to 13 for RosettaAntibody). On a target-by-target basis, Sphinx gave a more accurate prediction than RosettaAntibody 24 times, increasing to 28 when considering the best of the five top-ranked decoys.

In addition to achieving better accuracies, Sphinx is also much faster than RosettaAntibody. RosettaAntibody takes approximately 1 h to generate a single loop conformation (and hence up to 500 hours to generate the whole decoy set); Sphinx took (on average) 14 h per target to generate, minimize and rank all decoys, with the majority of the time being spent on minimization (approximately 12 h).

H3 loops can be classified as either ’kinked’ or ’extended’ depending on the conformation of their C-termini, with the majority being kinked (Weitzner *et al.*, 2015). Using the definition of Weitzner *et al.* (2015), we tested how often Sphinx correctly predicts a kinked or extended conformation. For the 30 kinked loops in the H3 target set, the top-ranked prediction of Sphinx was also kinked in 24 cases. This compares favourably to Rosetta, which only produced a top-ranked prediction of the correct conformation for 14 of the kinked targets. For both algorithms, five of the nine extended loops were predicted with the correct conformation.

We then used Sphinx to predict the H3 loops on antibody models rather than crystal structures, to see how accuracy is affected (Figure 6B). In the model environment, anchor residues are in a non-native conformation, making prediction more difficult. We generated model structures of the 39 antibodies in the set using ABodyBuilder (Leem *et al.*, 2016), a template-based fragment assembly method that builds antibody models using information from already known antibody structures. As expected, the results are not as good as when using the native environment – the average top-ranked RMSD increases from 2.5 (median 2.31 Å) to 3.26 Å (median 3.12 Å), and fewer sub-angstrom predictions are made. Interestingly, however, unlike the previous tests where minimization improved results, here it appears to decrease accuracy. The average top-ranked RMSD and the average RMSD of the best decoy in the five top-ranked stay approximately the same, but the number of targets predicted with an RMSD below 2 Å decreases from 18 to 10. In 2014, the second Antibody Modelling Assessment (AMA-II) was held; a CASP-style blind prediction test specifically for antibody structure prediction. The second round of this competition focused entirely on H3 prediction - participants were given the crystal structures of ten antibodies with the H3 loops removed, and asked to model the missing residues (allowing them to submit five possible models per target). We have used Sphinx to predict these H3 structures to further compare our results with those of other groups (all results are given in the Supplementary Information). We used the same protocol as that used for the previous H3 loop set, except that fragments from PDB structures deposited later than March 2013 were ignored (to ensure a fair comparison). We report our results using the same RMSD measure as used in AMA-II – using only the carbonyl C and O atoms of residues 95–100*x*.

On average, the best of the five models returned by Sphinx has an RMSD of 1.41 Å. This compares well to the other groups, who achieved RMSDs of 1.86, 2.09, 1.97, 1.25, 2.41 and 1.12 Å (for Accelrys, CCG, the Gray group, the Shirai group, Macromoltek and Schrödinger, respectively). Only the Shirai group and Schrödinger achieved better results than Sphinx according to this measure.

When looking at the average RMSD across all models, Sphinx outperforms all the other methods with an RMSD of 2.17 Å compared to 2.89, 3.08, 3.22, 2.43, 3.08 and 2.54 Å. Sphinx is therefore more consistent than the other methods. For two targets (Ab04 and Ab10), the best model from Sphinx had a lower or the same RMSD as the best produced from all other methods. In addition, Sphinx was able to produce a sub-angstrom prediction for six of the ten targets, which is the most achieved by any method (equal to the number generated by Schrödinger).

## 4 Conclusions

We have developed a loop modelling algorithm, Sphinx, which combines both knowledge-based and *ab initio* methodologies in a novel way, such that structural information contained within fragments of a different length to the target loop can be used. This is important, since it has been shown that loops of different lengths can adopt similar conformations (Nowak *et al.*, 2016), and other methods do not make use of this information. Sphinx is capable of generating high-quality decoy sets that are enriched with near-native conformations and in addition produces more accurate predictions than can be achieved using a straightforward *ab initio* algorithm. Sphinx is also able to produce a prediction for every target, unlike some knowledge-based methods, which can fail if a suitable fragment of the correct length cannot be found.

The H3-specific version of our algorithm, Sphinx-H3, produces promising results, achieving comparable or better results than some of the leading H3-specific *ab initio* algorithms (Teplyakov *et al.*, 2014). The kinked/extended conformation of the H3 targets was correctly predicted in the majority of cases – even though no prior assumptions or predictions were made about the loops, and no restraints were imposed on the decoys to force a kinked/extended structure. Sphinx is also fast – for example, in comparison to RosettaAntibody, Sphinx gives increased or similar accuracy in under 3% of the computation time. Sphinx is therefore a more viable option for use in applications such as structure-based virtual screening.

We have demonstrated that, except potentially in the case of modelling H3 in the non-native environment, the inclusion of a minimization procedure improves prediction accuracies, even though decoy RMSDs may not be consistently improved. This appears to be because the ability of the ranking method to correctly select near-native decoys is enhanced. However, this does not occur when predicting onto model structures – this could be because although the models onto which the decoys are built are good, they do not have atomic accuracy, and hence errors exist – especially in the conformations of the side chain. As such, the loop minimization is being performed in the non-native environment, which may affect results adversely. Preliminary results show that ranking is improved by minimizing the entire structure, not just the loop region.

The results achieved by our method indicate that fragments that are a slightly different length to the target loop are indeed a valuable source of structural information. Using a hybrid approach allows Sphinx to use the structural data that is available without being restricted to only returning loop conformations that have previously been observed. Also, since we have demonstrated that this approach can be applied successfully to prediction of the difficult H3 loop, we believe that the algorithm can be easily adapted for other specific protein loop types and should continue to perform well. For example, it could be applied to membrane protein loops, or the other five antibody CDR loops, since recent exploration into length-independent canonical conformations show that different length CDR loops can adopt the same conformation (Nowak *et al.*, 2016).

While Sphinx is the first algorithm of its kind, the concept behind it could easily be applied to other loop modelling algorithms, allowing them to use the information from structurally similar but length non-identical loops. For instance, in the case of antibody H3 prediction, RosettaAntibody could be combined with a knowledge-based method [such as FREAD or H3Loopred (Messih *et al.*, 2014)] to offer an alternative.

From the results presented in this paper, we believe that in a real loop modelling situation a knowledge-based method such as FREAD should be tried first (since such methods are usually very fast). However, if a suitable fragment cannot be found, then the next approach should be to use a hybrid algorithm such as Sphinx. Instead of reverting to a pure *ab initio* algorithm that ignores previously observed structures, this allows more of the available structural data to be used, leading to more accurate predictions.

As the results presented here demonstrate that, using the extra structural information contained within different length loops, Sphinx produces high-quality decoy sets and accurate predictions in a fraction of the time of some existing algorithms. As the PDB expands and more structural data becomes available, Sphinx’s performance should improve further.

## Funding

This work was supported by the Engineering and Physical Sciences Research Council (grant reference EP/G037280/1), UCB Pharma Ltd and Roche.

*Conflict of Interest*: none declared.

## Supplementary Material

Supplementary Data
