# Supplementary material for: Sphinx: merging knowledge-based and ab initio approaches to improve protein loop prediction
Source: Bioinformatics. 2017 Jan 16;33(9):1346–53. doi: 10.1093/bioinformatics/btw823 (PMC5408792; doi:10.1093/bioinformatics/btw823)
Supplement: Supplementary Data [file btw823_supp.pdf]

Supplementary Information

Contents:

1. Colour Version of Figure 1 - Sphinx Algorithm Flowchart
2. Modified CCD Algorithm
3. Rosetta Minimisation
4. Testing Ranking Methods
5. Resampled H3 Ramachandran distributions
6. H3 Targets Used During Exploratory Testing
7. Colour Version of Figure 2 - Sphinx Results
8. Algorithm Commands
9. Colour Version and Full Results for Figure 5 - Comparison to MODELLER and RAPPER
10. AMA-II Set Results

1 Colour Version of Figure 1 - Sphinx Algorithm Flowchart

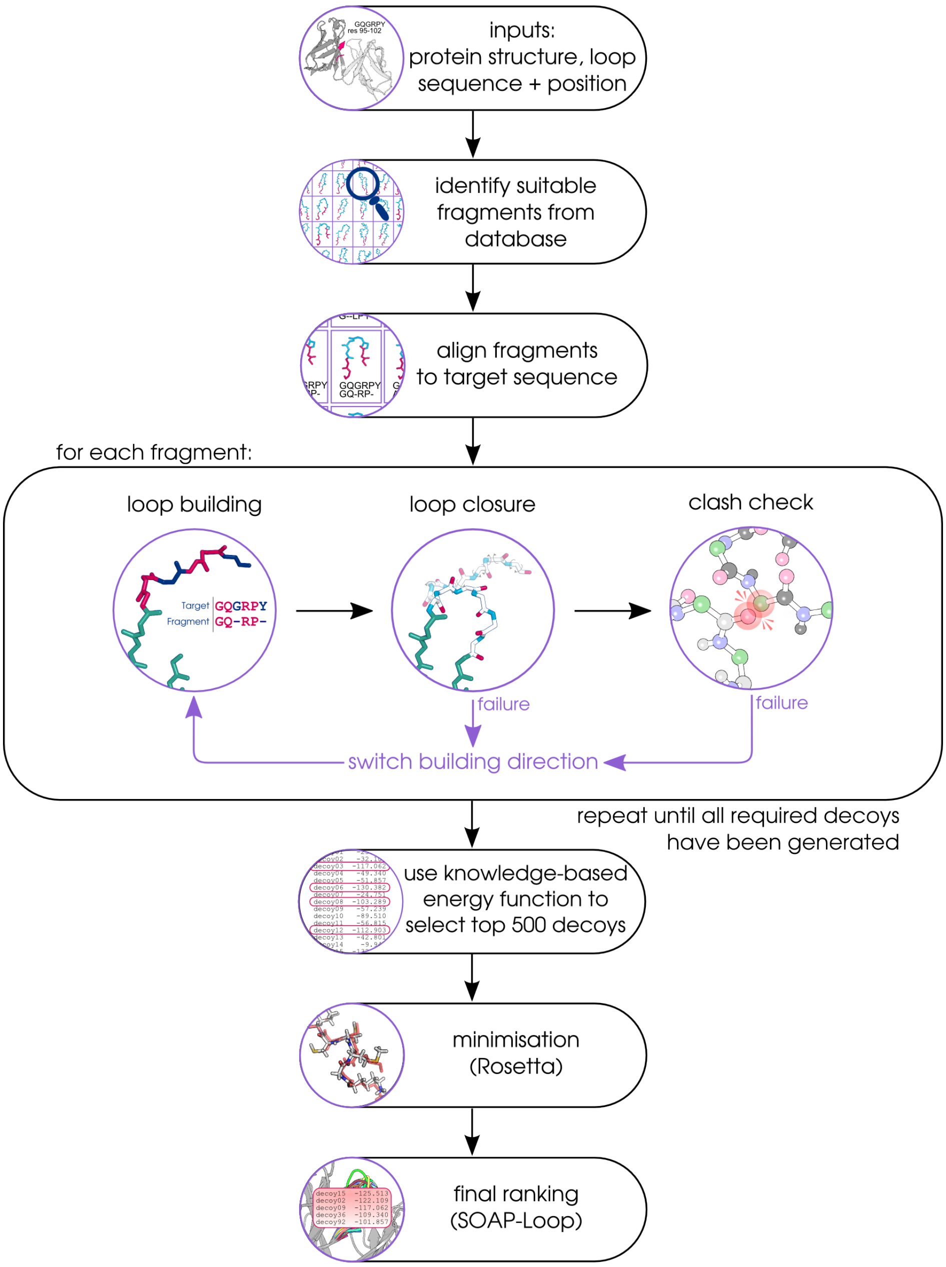

Figure 1: Flowchart describing the Sphinx algorithm. As input, the algorithm requires the protein structure on which the loop is to be modelled, and the loop sequence and location. The algorithm begins by searching through a database of loop fragments. Suitable fragments, that are shorter than the target loop, are used to help build a set of 100 decoys. The number of fragments used depends on the length (n) of the target - $12.5(n-2)$ for even lengths and $12.5(n-1)$ for odd lengths. For any residues aligned between the target and the fragment, structural information is copied across. Any residues not aligned to fragment residues are built *ab initio*. Each loop structure undergoes a closure step and a clash check - if closure fails or there are clashes, the next decoy is built from the alternate anchor residue ('switching'). Once all decoys have been generated, a loop-specific energy function is used to select the top 500 decoys, which subsequently undergo minimisation (using Rosetta) and final ranking (with SOAP-Loop).

2 Modified CCD Algorithm

Using the ϕ/ψ data described in Section 2.2 of the paper, we calculated probabilities for all possible ϕ/ψ combinations. For every residue type, the resampled data was split into 5° bins, and the probability for each bin was calculated by dividing the number of points found in each bin by the total number of points. In the same way as the normal CCD algorithm, residues are considered sequentially, except the ϕ/ψ angles are now considered in pairs. The changes in the angles required to minimise the distance between the free end of the loop and the anchor residue is calculated, along with the probabilities of both the current ϕ/ψ angles and the new ones. If the probability of the new angle pair is greater than the previous one, then the angle change is accepted; however if it is less, the change is accepted with a probability given by the probability of observing the proposed angles divided by the probability of observing the current angles. The algorithm is therefore restricted from changing the dihedral angles to disallowed values.

3 Rosetta Minimisation

To minimize the loop structures we have applied short runs of the Next-Generation Kinematic closure (NGK) protocol implemented in Rosetta (Mandell *et al.*, 2009). Briefly, the algorithm designates three residues as pivots. Next, new values for the torsions of the remaining residues are sampled, opening the loop. Finally, the algorithm searches for torsion values for the pivotal residues that close the loop. These steps are iterated in a Monte Carlo framework to search for a conformation that minimizes the Rosetta energy.

Mandell, D. J., Coutsias, E.A. and Kortemme, T. (2009). Sub-angstrom accuracy in protein loop reconstruction by robotics-inspired conformational sampling. *Nat. Methods*, **6**(8), 551-2.

4 Testing Ranking Methods

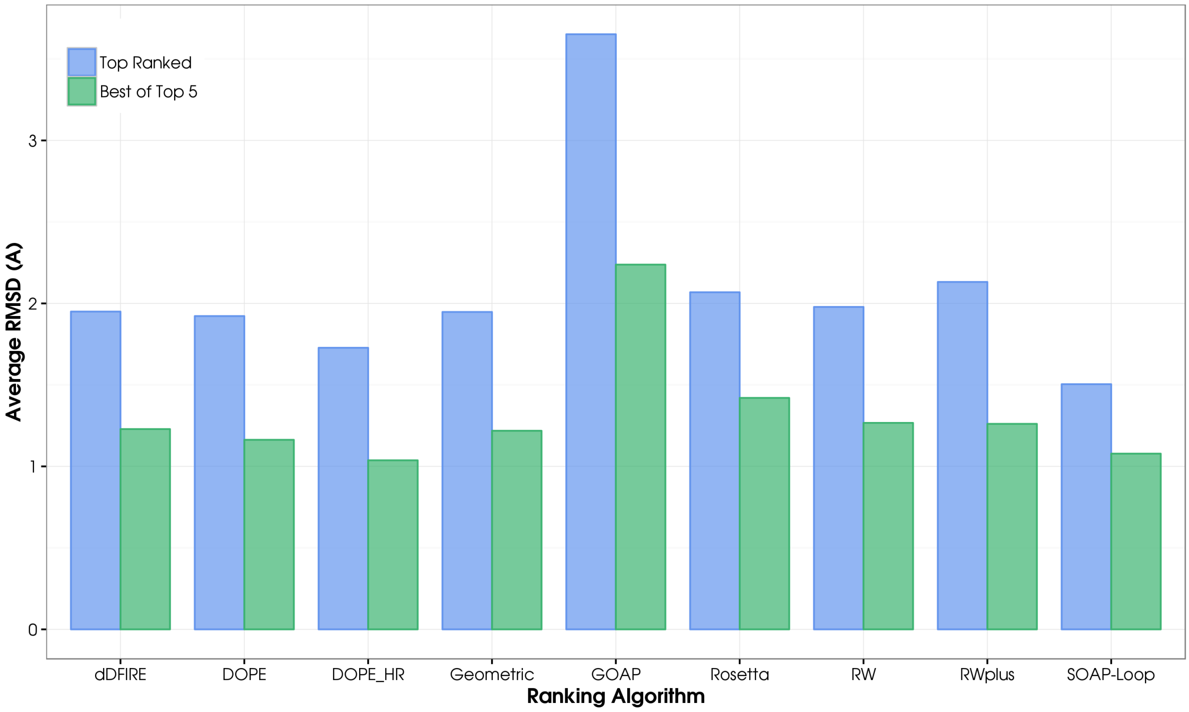

Figure 2: Average RMSDs of the top ranked decoy and the best of the top 5 decoys, as given by different ranking methods.

References:

dDFIRE - Yang, Y. and Zhou, Y. (2008). Specific interactions for ab initio folding of protein terminal regions with secondary structures. *Proteins*, **72**(2), 793-803.

DOPE/DOPE_HR - Shen, M. and Sali, A. (2006). Statistical potential for assessment and prediction of protein structures. *Protein Sci.*, **15**(11), 2507-2524.

Geometric - Li, X. and Liang, J. (2007). Geometric packing potential function for model selection in protein structure and protein-protein binding predictions. Unpublished. Downloaded from http://gila.bioe.uic.edu/resources/geometric.html

GOAP - Zhou, H. and Skolnick, J. (2011). GOAP: a generalized orientation-dependent, all-atom statistical potential for protein structure prediction. *Biophys. J.*, **101**(8), 2043-2052.

Rosetta - O'Meara, M. J., Leaver-Fay, A., Tyka, M. D., Stein, A., Houlihan, K., DiMaio, F., Bradley, P., Kortemme, T., Baker, D., Snoeyink, J. and Kuhlman, B. (2015). Combined covalent-electrostatic model of hydrogen bonding improves structure prediction with Rosetta. *J. Chem. Theory Comput.*, **11**(2), 609-622.

RW/RWplus - Zhang, J. and Zhang, Y. (2010). A novel side-chain orientation dependent potential derived from random-walk reference state for protein fold selection and structure prediction. *PLoS ONE*, **5**(10), e15386.

SOAP-Loop - Dong, G. Q., Fan, H., Schneidman-Duhovny D., Webb, B. and Sali, A. (2013). Optimized atomic statistical potentials: assessment of protein interfaces and loops. *Bioinformatics*, **29**(24), 3158-3166.

5 Resampled H3 Ramachandran Distributions

As there are currently not enough H3 structures available to produce residue-specific Ramachandran distributions, we constructed the distributions using a novel resampling method that combines general protein loop and H3 data. H3 dihedral data was obtained by calculating the ϕ/ψ angles of all the H3 loops in SAbDab. General protein loop data was extracted from a set of protein structures (obtained using PISCES) meeting the following criteria: non-redundancy (maximum sequence identity of 60%); resolution below 2 Å; R factor below 0.3. Loop regions were extracted using the secondary structure definitions given by DSSP. For each amino acid type, random ϕ/ψ angles were chosen from the general loop data and added to the H3 distributions, with a probability dependent on the proximity of the random angles to those already present in the H3 data. The nearest point in the H3 distribution is found (using a K-nearest neighbour search algorithm), and the probability of addition is calculated using a Gaussian function centred on this point with a standard deviation of $\sqrt{8}$. If the randomly chosen point from the general loop distribution is more than one standard deviation from its nearest neighbour in the H3 distribution, then those angles are discarded. If the random point already exists in the H3 distribution, then it is added with a probability of 1. Once a point has been added to the distribution, it can then be considered as a nearest neighbour for the following resampling iterations. A total of 20,000 iterations were carried out to give the final resampled distributions.

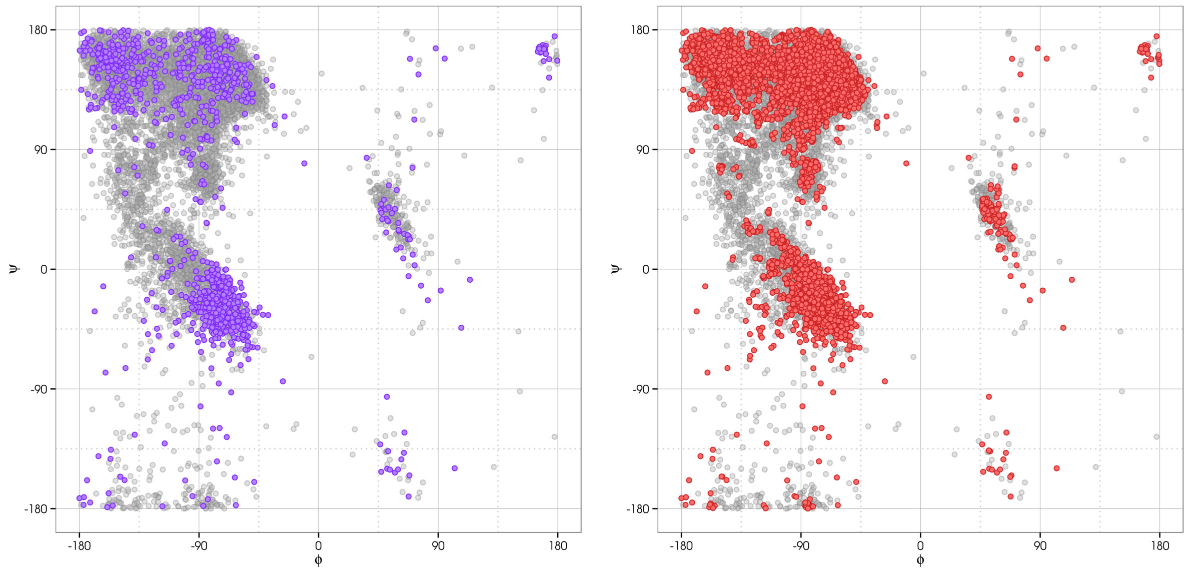

Figure 3: Ramachandran resampling, shown for alanine residues. H3 ψ/ϕ dihedral angles (purple, left plot) are different to general protein ψ/ϕ dihedral angles (grey, both plots), but there is a lack of H3 data. By using our resampling method, we can generate a Ramachandran distribution (red, right plot) that is less sparse than the original H3 data, but maintains its unique nature.

6 H3 Targets Used During Exploratory Testing

PDB	Length	Sequence	PDB	Length	Sequence	PDB	Length	Sequence
4hxbH	6	LRAVDY	1blnB	10	YYRYEAWFAS	4dgyH	14	DISLVRDAFIYFDF
1za6B	6	SLNMAY	4g6mH	10	NRYDPPWFVD	1fnsH	14	DPADYGNYDYALDY
1z3gH	6	GWDVAY	1jn6B	10	GRSLYYTMDY	2hrpH	16	SGGIERYDGTYYVMDY
3cfbB	6	GQGRPY	1eapB	10	SYYGSSYVDY	3qehA	16	DLEMRDGNNHGSHLEF
2uziH	6	GRFFDY	3wihH	10	FSNYVYPFDY	4fqjH	16	DVQYSGSYLGAYYFDY
1igyB	6	EGEVPY	2vl5A	10	LKPGGTWFAY	4olzH	16	GKYCTARDYYNWDFEH
3d85B	6	NWDVAY	1jrhH	12	RAPFYGNHAMDY	1etzB	16	RTFSYYYGSSFYYFDN
1f3dH	6	DYDGVY	2g5bB	12	DNGSDYRWYFDV	4grwE	16	VRTGWGLNAPDYAMDY
1bafH	6	GWPLAY	1kfaH	12	EGLLLDYFTMDY	4olvH	16	GKYCTARDYYNWDFEH
1sm3H	6	VGQFAY	3bgfB	12	EGIPQLLRTLDY	3lmjH	16	GGNYYNLWTGYYPLAY
1mimH	8	DYGYYFDF	3rvvD	12	TGVYRYPERAPY	2xqbH	16	DPAAWPLQQSLAWFDP
2pcpB	8	STWDDFDY	2vxvH	12	VGSGWYPYTFDI	3qycA	16	LPDLCPGDNCTYPDAS
1b4jH	8	GFLPWFAD	1mnuH	12	IYFDYADFIMDY	2x1pA	18	DLHRPYGPGTQRSDEYDS
1a6tB	8	RDDYYFDF	4rgmC	12	IYYGNNGGVMDY	4b5eA	18	REGDVGLVSYKRSSNYPY
3hr5B	8	DNWDAMDY	3eo9H	12	GIYFYGTTYFDY	3r0mA	18	KWRPLRYSDYPSNSDYYD
4o4yH	8	GLYNDYTV	3i9gH	12	GGFYGSTIWFDF	3grwH	18	TYGIYDLYVDYTEYVMDY
1a7qH	8	ERDYRLDY	2qqnH	14	GELPYYRMSKVMDV	1zvyA	18	TRKYVPVRFALDQSSYDY
3uolH	8	LTNGYLDV	1yejH	14	WGFIPVREDYVMDY	4dkaA	18	DRFPTMEVVTIMTNEYDY
4lkxA	8	MGYDGLAY	4nbxB	14	APYGANWYRDEYAY	4i13B	18	SRISYRVWNTIPYNKLTL
1kelH	8	WGSYAMDY	2dquH	14	VSHYDGSRDWYFDV	2fx7H	18	EGTTGWGWLGKPIGAFAH
1rurH	10	AGGYYTGGDY	3rkdD	14	IKSVITTGDYALDY	1n0xH	18	VGPYSWDDSPQDNYYMDV
4ojfH	10	YDHYSGSSDY	2brrH	14	DYYGSTYPYYAMDY	2x1qA	18	SSRVFYTEVLQTTTGYDY
1ucbH	10	GLDDGAWFAY	1kxqE	14	GNSVRLASWEGYFY			
4gagH	10	ITTTTYAMDY	4grwH	16	VRTGWGLNAPDYAMDY			

Table 1: The set of targets used to verify the ability of Sphinx to predict H3 loop structures. The residues included in the H3 loop are in accordance with the Chothia definition.

7 Colour Version of Figure 2 - Sphinx Results

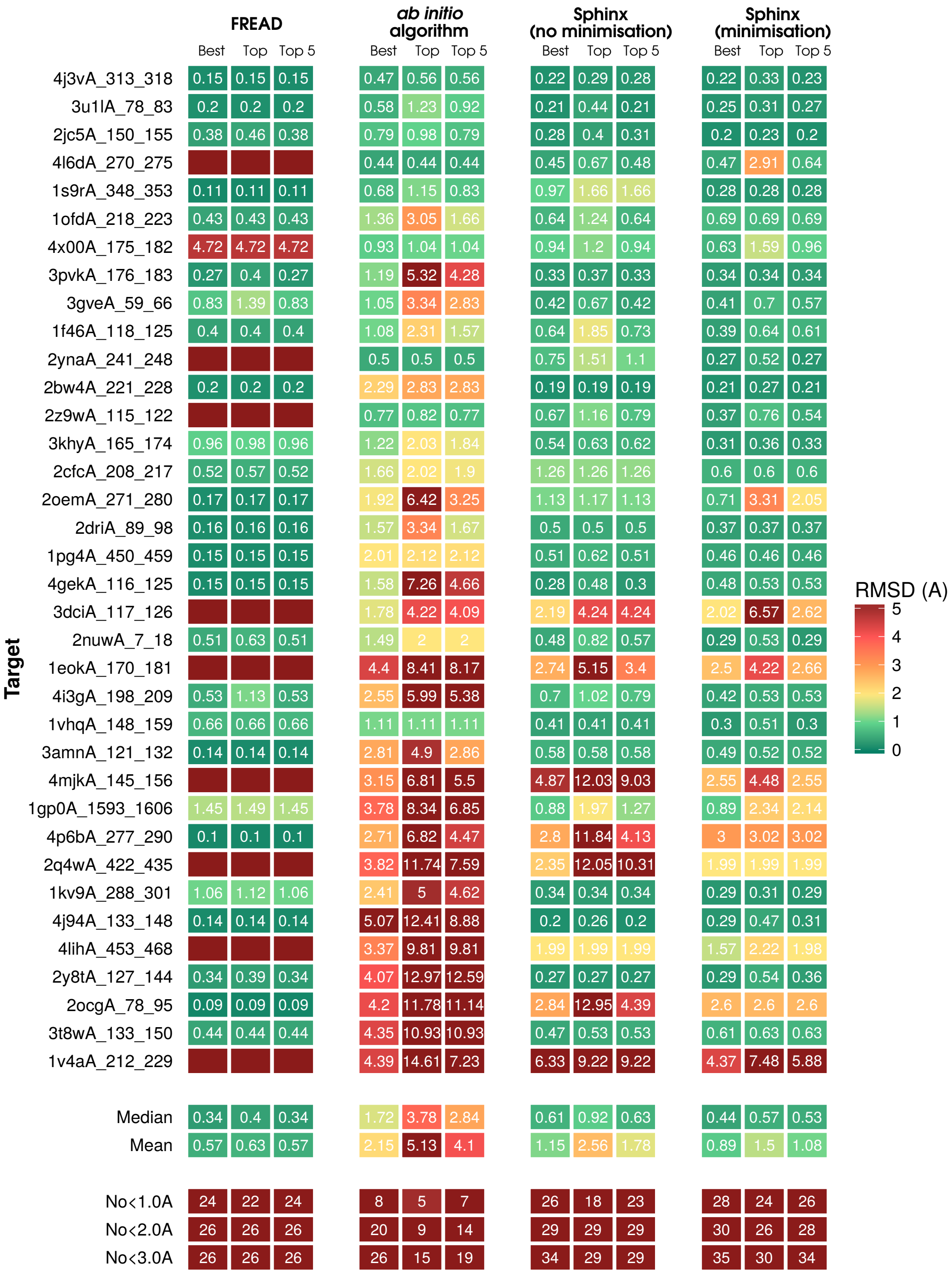

Target	FREAD Best	FREAD Top	FREAD Top 5	*ab initio* algorithm Best	*ab initio* algorithm Top	*ab initio* algorithm Top 5	Sphinx (no minimisation) Best	Sphinx (no minimisation) Top	Sphinx (no minimisation) Top 5	Sphinx (minimisation) Best	Sphinx (minimisation) Top	Sphinx (minimisation) Top 5
4j3vA_313_318	0.15	0.15	0.15	0.47	0.56	0.56	0.22	0.29	0.28	0.22	0.33	0.23
3u1lA_78_83	0.2	0.2	0.2	0.58	1.23	0.92	0.21	0.44	0.21	0.25	0.31	0.27
2jc5A_150_155	0.38	0.46	0.38	0.79	0.98	0.79	0.28	0.4	0.31	0.2	0.23	0.2
4l6dA_270_275				0.44	0.44	0.44	0.45	0.67	0.48	0.47	2.91	0.64
1s9rA_348_353	0.11	0.11	0.11	0.68	1.15	0.83	0.97	1.66	1.66	0.28	0.28	0.28
1ofdA_218_223	0.43	0.43	0.43	1.36	3.05	1.66	0.64	1.24	0.64	0.69	0.69	0.69
4x00A_175_182	4.72	4.72	4.72	0.93	1.04	1.04	0.94	1.2	0.94	0.63	1.59	0.96
3pvkA_176_183	0.27	0.4	0.27	1.19	5.32	4.28	0.33	0.37	0.33	0.34	0.34	0.34
3gveA_59_66	0.83	1.39	0.83	1.05	3.34	2.83	0.42	0.67	0.42	0.41	0.7	0.57
1f46A_118_125	0.4	0.4	0.4	1.08	2.31	1.57	0.64	1.85	0.73	0.39	0.64	0.61
2ynaA_241_248				0.5	0.5	0.5	0.75	1.51	1.1	0.27	0.52	0.27
2bw4A_221_228	0.2	0.2	0.2	2.29	2.83	2.83	0.19	0.19	0.19	0.21	0.27	0.21
2z9wA_115_122				0.77	0.82	0.77	0.67	1.16	0.79	0.37	0.76	0.54
3khyA_165_174	0.96	0.98	0.96	1.22	2.03	1.84	0.54	0.63	0.62	0.31	0.36	0.33
2cfcA_208_217	0.52	0.57	0.52	1.66	2.02	1.9	1.26	1.26	1.26	0.6	0.6	0.6
2oemA_271_280	0.17	0.17	0.17	1.92	6.42	3.25	1.13	1.17	1.13	0.71	3.31	2.05
2driA_89_98	0.16	0.16	0.16	1.57	3.34	1.67	0.5	0.5	0.5	0.37	0.37	0.37
1pg4A_450_459	0.15	0.15	0.15	2.01	2.12	2.12	0.51	0.62	0.51	0.46	0.46	0.46
4gekA_116_125	0.15	0.15	0.15	1.58	7.26	4.66	0.28	0.48	0.3	0.48	0.53	0.53
3dciA_117_126				1.78	4.22	4.09	2.19	4.24	4.24	2.02	6.57	2.62
2nuwA_7_18	0.51	0.63	0.51	1.49	2	2	0.48	0.82	0.57	0.29	0.53	0.29
1eokA_170_181				4.4	8.41	8.17	2.74	5.15	3.4	2.5	4.22	2.66
4i3gA_198_209	0.53	1.13	0.53	2.55	5.99	5.38	0.7	1.02	0.79	0.42	0.53	0.53
1vhqA_148_159	0.66	0.66	0.66	1.11	1.11	1.11	0.41	0.41	0.41	0.3	0.51	0.3
3amnA_121_132	0.14	0.14	0.14	2.81	4.9	2.86	0.58	0.58	0.58	0.49	0.52	0.52
4mjkA_145_156				3.15	6.81	5.5	4.87	12.03	9.03	2.55	4.48	2.55
1gp0A_1593_1606	1.45	1.49	1.45	3.78	8.34	6.85	0.88	1.97	1.27	0.89	2.34	2.14
4p6bA_277_290	0.1	0.1	0.1	2.71	6.82	4.47	2.8	11.84	4.13	3	3.02	3.02
2q4wA_422_435				3.82	11.74	7.59	2.35	12.05	10.31	1.99	1.99	1.99
1kv9A_288_301	1.06	1.12	1.06	2.41	5	4.62	0.34	0.34	0.34	0.29	0.31	0.29
4j94A_133_148	0.14	0.14	0.14	5.07	12.41	8.88	0.2	0.26	0.2	0.29	0.47	0.31
4lihA_453_468				3.37	9.81	9.81	1.99	1.99	1.99	1.57	2.22	1.98
2y8tA_127_144	0.34	0.39	0.34	4.07	12.97	12.59	0.27	0.27	0.27	0.29	0.54	0.36
2ocgA_78_95	0.09	0.09	0.09	4.2	11.78	11.14	2.84	12.95	4.39	2.6	2.6	2.6
3t8wA_133_150	0.44	0.44	0.44	4.35	10.93	10.93	0.47	0.53	0.53	0.61	0.63	0.63
1v4aA_212_229				4.39	14.61	7.23	6.33	9.22	9.22	4.37	7.48	5.88
Median	0.34	0.4	0.34	1.72	3.78	2.84	0.61	0.92	0.63	0.44	0.57	0.53
Mean	0.57	0.63	0.57	2.15	5.13	4.1	1.15	2.56	1.78	0.89	1.5	1.08
No<1.0A	24	22	24	8	5	7	26	18	23	28	24	26
No<2.0A	26	26	26	20	9	14	29	29	29	30	26	28
No<3.0A	26	26	26	26	15	19	34	29	29	35	30	34

Figure 4: Performance of Sphinx compared to its constituent knowledge-based and *ab initio* parts, for general protein loop types. Targets are listed in order of increasing length. 'Best' refers to the decoy in the set that is closest to the native structure, when considering the 500 decoys returned by the loop-specific energy function (for Sphinx and the *ab initio* method) or all the returned decoys (for FREAD). 'Top' means the decoy that is predicted to be the best, and 'Top 5' denotes the RMSD of the best decoy amongst the five top-ranked.

8 Algorithm Commands

RAPPER results were obtained using the following command:

```
rapper_Linux_i386 /path/to/params_Linux_i386.xml model-loops-benchmark
   --rapper-dir <location of RAPPER executables> --pdb <pdb file> --chain-id <chain ID>
   --models 1000 --start <start residue> --stop <end residue> --runs-dir <results directory>
```

The results achieved for the loop modelling algorithm within MODELLER were obtained using the Python `modeller.automodel` module, according to the instructions given on the MODELLER website (salilab.org/modeller/wiki/Missing%20residues):

```
class MyModel(automodel):
   def select_atoms(self):
      return selection(self.residue_range(<start residue>, <end residue>))

a = MyModel(env, <alignment file>, knowns = <pdb file with missing loop>,
   sequence = <loop sequence id>, assess_methods=assess.DOPE)
a.starting_model = 1
a.ending_model = 500
a.make()
```

The H3 prediction results achieved by RosettaAntibody (version 2016.11.58563) were obtained using the following command:

```
antibody_H3.static.linuxgccrelease -s <pdbfile> -antibody::remodel perturb_kic
   -antibody::snugfit true -antibody::refine refine_kic -antibody::cter_insert false
   -antibody::flank_residue_min true -antibody::bad_nter false
   -antibody::h3_filter true -antibody::h3_filter_tolerance 20
   -antibody:constrain_vlvh_qq -nstruct 1 -ex1 -ex2 -extrachi_cutoff 0
   -kic_bump_overlap_factor 0.36 -corrections:score:use_bicubic_interpolation false
   -loops:legacy_kic false -loops:kic_min_after_repack true
   -loops:kic_omega_sampling -loops:allow_omega_move true
   -loops:ramp_fa_rep -loops:ramp_rama -loops:kic_rama2b
```

9 Colour Version and Full Results for Figure 5 - Comparison to MODELLER and RAPPER

Target	Sphinx Best	Sphinx Top	Sphinx Top 5	MODELLER Best	MODELLER Top	MODELLER Top 5	RAPPER (1) Best	RAPPER (1) Top	RAPPER (1) Top 5	RAPPER (2) Best	RAPPER (2) Top	RAPPER (2) Top 5
4j3vA_313_318	0.22	0.33	0.23	0.82	0.82	0.82	1.19	3.89	3.1	0.73	0.98	0.8
3u1lA_78_83	0.25	0.31	0.27	0.94	3.37	2.76	0.53	1.53	1.38	0.47	0.47	0.47
2jc5A_150_155	0.2	0.23	0.2	0.8	0.99	0.9	0.92	2.01	1.82	0.46	1.09	1.09
4l6dA_270_275	0.47	2.91	0.64	1.11	1.24	1.24	0.83	1.8	1.8	0.95	1.55	1.4
1s9rA_348_353	0.28	0.28	0.28	0.63	0.7	0.63	0.63	2.36	2.36	0.44	0.44	0.44
1ofdA_218_223	0.69	0.69	0.69	1.75	2.23	1.86	1.6	3.32	2.71	1.32	1.5	1.41
4x00A_175_182	0.63	1.59	0.96	2.07	2.07	2.07	1.26	3.14	3.12	1.03	1.66	1.49
3pvkA_176_183	0.34	0.34	0.34	3.87	7.52	5.16	1.58	7.22	5.69	1.31	2.11	1.6
3gveA_59_66	0.41	0.7	0.57	3.04	3.25	3.17	1.16	2.3	1.36	1.15	2.23	1.37
1f46A_118_125	0.39	0.64	0.61	0.99	0.99	0.99	1.75	4.19	2.27	1.04	1.15	1.04
2ynaA_241_248	0.27	0.52	0.27	1.43	1.74	1.71	0.98	1.86	1.6	0.64	0.74	0.74
2bw4A_221_228	0.21	0.27	0.21	2.73	4.84	2.97	1.25	6.38	3.86	1.04	1.04	1.04
2z9wA_115_122	0.37	0.76	0.54	1.16	2.74	2.74	2.33	4.37	4.37	0.86	1.23	1.23
3khyA_165_174	0.31	0.36	0.33	4.25	4.25	4.25	0.96	3.03	2.21	0.62	0.62	0.62
2cfcA_208_217	0.6	0.6	0.6	1.38	2.38	1.65	1.39	7.01	2.75	1.07	1.35	1.35
2oemA_271_280	0.71	3.31	2.05	5.85	6.48	6.48	1.98	7.16	6.17	2.2	7.1	6.41
2driA_89_98	0.37	0.37	0.37	10.6	10.92	10.92	1.44	4.76	4.76	1.23	1.58	1.5
1pg4A_450_459	0.46	0.46	0.46	2.16	4.3	2.31	2.69	9.88	5.39	3.59	8.61	4.89
4gekA_116_125	0.48	0.53	0.53	2.79	2.87	2.79	1.71	6.15	4.42	1.44	1.54	1.44
3dciA_117_126	2.02	6.57	2.62	2.63	4.07	3.24	2.56	7.58	5.03	2.32	7.72	2.46
2nuwA_7_18	0.29	0.53	0.29	1.55	4.61	2.24	1.79	2.37	2.37	0.77	1.73	0.77
1eokA_170_181	2.5	4.22	2.66	5.84	8.11	7.96	3.08	5.68	5.68	2.2	3.32	2.8
4i3gA_198_209	0.42	0.53	0.53	13.33	14.31	13.94	1.63	2.72	2.72	2.03	4.15	2.31
1vhqA_148_159	0.3	0.51	0.3	1.45	4.14	2.92	2.08	11.11	2.93	2.96	9.84	4.33
3amnA_121_132	0.49	0.52	0.52	2.67	5.63	4.12	2.04	9.93	8.22	2.68	5.9	3.18
4mjkA_145_156	2.55	4.48	2.55	7.96	10.53	7.98	2.98	8.69	8.69	3.79	7.28	7.14
1gp0A_1593_1606	0.89	2.34	2.14	4.34	4.34	4.34	2.63	9.79	6.92	4.13	15.14	8.41
4p6bA_277_290	3	3.02	3.02	3.9	4.15	4.15	2.89	8.3	7.37	4.13	5.1	5.1
2q4wA_422_435	1.99	1.99	1.99	6.59	6.59	6.59	5.55	12.44	11.39	3.02	3.56	3.02
1kv9A_288_301	0.29	0.31	0.29	9.92	11.71	11.38	1.64	2.67	2.67	1.04	1.13	1.04
4j94A_133_148	0.29	0.47	0.31	7.83	14.85	12.61	6.07	10.4	10.4	7.01	10.49	9.14
4lihA_453_468	1.57	2.22	1.98	11.98	12.7	12.7	6.01	10.52	8.73	5.01	11.11	6.59
2y8tA_127_144	0.29	0.54	0.36	11.74	15.97	12.34	3.91	12.09	9.14	3.73	6.21	3.73
2ocgA_78_95	2.6	2.6	2.6	7.02	16.25	14.9	3.27	11.6	7.92	4.87	11.05	5.69
3t8wA_133_150	0.61	0.63	0.63	3.04	4.77	4.47	5.91	9.3	8.45	5.58	12.06	7.92
1v4aA_212_229	4.37	7.48	5.88	6.09	10.83	8.96	4.29	10.82	10.82	5.61	8.75	6.83
Median	0.44	0.57	0.53	2.92	4.32	3.68	1.77	6.27	4.39	1.38	2.17	1.55
Mean	0.89	1.5	1.08	4.34	6.03	5.28	2.35	6.34	5.02	2.29	4.49	3.08
No<1.0A	28	24	26	5	4	4	6	0	0	9	5	6
No<2.0A	30	26	28	12	6	8	20	3	5	19	17	19
No<3.0A	35	30	34	18	11	16	28	9	14	25	19	22

RMSD (A): 0 1 2 3 4 5

Figure 5:

10 AMA-II Set Results

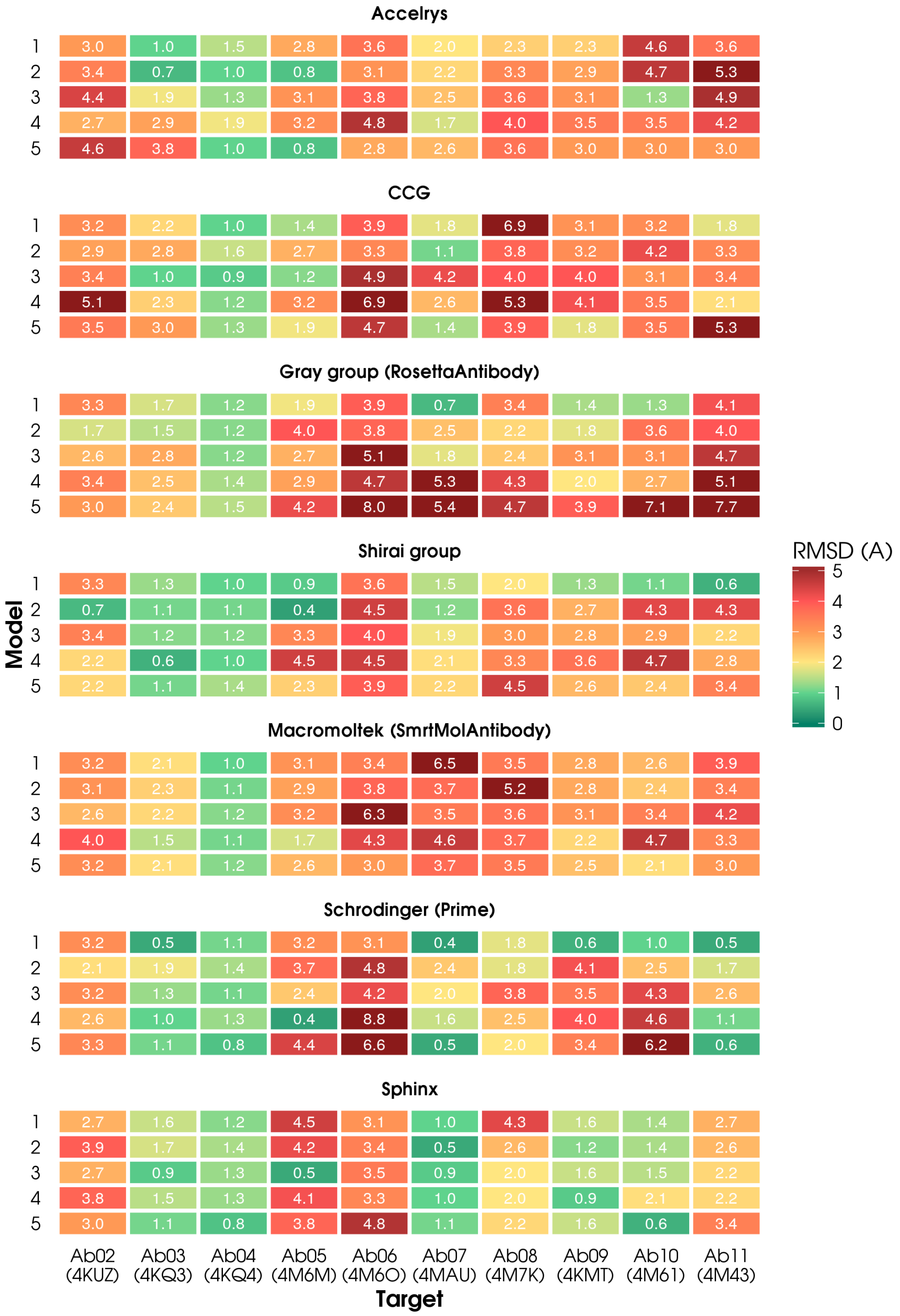

Figure 6: Results of using Sphinx to predict the H3 loops in the AMA-II set, compared to those achieved by the participants of the second round of the second Antibody Modelling Assessment. RMSDs reported are calculated over the carbonyl C and O atoms of residues 95-100x.
